# Arsenic, Cadmium and Lead Exposure and Immunologic Function in Workers in Taiwan

**DOI:** 10.3390/ijerph15040683

**Published:** 2018-04-05

**Authors:** Chin-Ching Wu, Fung-Chang Sung, Yi-Chun Chen

**Affiliations:** 1 Department of Public Health, China Medical University, 91 Hsueh-Shih Road, Taichung 404, Taiwan; wucc@mail.cmu.edu.tw; 2 Department of Health Services Administration, China Medical University, 91 Hsueh-Shih Road, Taichung 404, Taiwan; fcsung1008@yahoo.com; 3 Department of Health Management, College of Medicine, I-Shou University, 8 Yida Road, Yanchao District, Kaohsiung 824, Taiwan

**Keywords:** arsenic, cadmium, lead, hair, immunologic function

## Abstract

There has been growing concern over the impact of environmental exposure to heavy metals and other trace elements on immunologic functions. This study investigated men’s arsenic (As), cadmium (Cd) and lead (Pb) contents in hair samples and their associations with immunological indicators, including white blood cell (WBC), lymphocyte and monocyte counts, and the immunoglobulin (Ig) levels including IgA, IgG and IgE. We recruited 133 men from one antimony trioxide manufacturing plant, two glass manufacturing plants and two plastics manufacturing plants. The mean concentration of Cd [0.16 (SD = 0.03) ug/g] was lower than means of As [0.86 (SD = 0.16) ug/g] and Pb [0.91 (SD = 0.22) ug/g] in hair samples, exerting no relationship with immunologic functions for Cd. The Spearman’s correlation analysis showed a positive relationship between monocyte counts and hair Pb levels, but negative relations between As and IgG and between As and IgE. In conclusion, findings from these industry workers suggest that As levels in hair may have a stronger relation with immunologic function than Cd and PB have. Further research is needed to confirm the negative relationship.

## 1. Introduction

Studies have found that exposure to heavy metals and other trace elements may compromise various aspects of the immune system. Among these elements, arsenic (As), cadmium (Cd) and lead (Pb) have received more attention and investigations [1,2,3,4,5,6]. Cell culture study shows that the chemotherapeutic agent arsenic trioxide could inhibit early T cell cytokine production. [7]. Negative associations have been found between urine arsenic levels and proliferative response to phytohemaglutinin stimulation, CD4, CD4/CD8 ratio, and IL-2 secretion levels in children [1]. In an animal study, rats consuming water with 50.0 ppm of Cd for 30 days are at greater risk of intestinal inflammation than those with 5.0 ppm of Cd [3]. Consuming a high amount of Cd increases the numbers of NK and CD68 (+) cells, oxidative activities and IL-1β. Lead is a ubiquitous environmental pollutant posing a serious health threat to humans. Blood lead levels below 10 μg/dL may exert the later-life immune alteration by reducing the Type 1T helper cell response and elevating the autoimmune risk [5]. 

Studies on relationships between heavy metal exposure and immunological effect using in vivo and in vitro methods are with conflicting results [2,8]. Limited studies have observed effects of trace elements on humans using immunologic indicators. The cross-sectional study using National Health and Nutrition Examination Survey (NHANES) data from 1999 to 2012 showed that elevated blood levels of Pb and Cd might increase the susceptibility to chronic infections [6]. Workers with higher Pb exposure may have depressed T helper lymphocytes, IgG, IgM and C3, C4 complement levels, chemotaxis, and random migration of neutrophils [9]. These relationships for humans with low metal exposures have not been fully confirmed. This study aims to examine men’s blood As, Cd and Pb levels and their associations with immunological indicators among industrial workers. 

## 2. Materials and Methods 

### 2.1. Sample Collection and Treatment

This study recruited 133 male employees at one plant manufacturing antimony trioxide, two plants manufacturing glasses and two plants manufacturing plastics. Our study designs and procedures have been reported previously [10]. In brief, we collected specimens of blood, urine and hair from each participant with consent after the IRB approval (DMR99-IRB-142(FR)). Digested samples were analyzed using Inductively Coupled Plasma Mass Spectrometer (ICP-MS) (Perkin-Elmer SCIEX ELAN DRC II, Concord, ON, Canada) by an analyzer who was blinded to sample identifications. Blood samples were also collected for the assay of white blood cell (WBC) count, lymphocyte and monocyte and the immunoglobulin assay, including IgA, IgG and IgE. Information on socio-demographic status, lifestyle and work history were collected using questionnaires. 

For the present study, we used As, Cd and Pb measured from hair samples to evaluate the relationships with immunologic factors. Because of slow growth, elements measured in hair represent a longer accumulation of the elemental exposure from diet, drink and air [11,12,13]. The hair sample analysis thus has an advantage over blood and urine samples, which show the current and recent burden. We considered that trace elements in hair could be better representative of exposure. Although, findings from hair mineral analysis may vary among laboratories [14]. A pinch of hairs near the neck from each participant was collected for analyzing trace elements. We used 1:200 (*v*/*v*) Triton X-100 solutions to clean hair samples, followed by acetone, and washed samples twice with deionized water. Clean samples were dried at 75 °C for 24 h in an oven, then stored in an electronic dry cabinet at room temperature for 12 h or longer until digestion. The dried hair (0.2 g) was digested in a microwave with 3 mL of 70% nitric acid and diluted to 10 mL with 1% (*v*/*v*) hydrochloric acid. The analyzer mixed 1 mL of the solution, 1 mL of Indium standard solution (as an internal standard) and 8 mL of 1% (*v*/*v*) hydrochloric acid to measure trace elements, using ICP-MS (Perkin-Elmer SCIEX ELAN DRC II, Waltham, MA, USA) [15]. The limit of detection (LOD) of ICP-MS for blanks and the limit of quantification (LOQ) for the hair samples were 0.0008 µg/g and 0.014 µg/g for Pb, 0.0000 µg/g and 0.018 µg/g for Cd and 0.0015 µg/g and 0.021 µg/g for As, respectively. For further validating the analytical method, we conducted external quality control analyses to measure concentrations of these 3 elements using a certified reference hair sample (CRM GBW-09101-Human Hair, Shanghai Institute of Nuclear Research Academia Sinica, Shanghai, China). The recovery rates for the 3 elements were 91.2%, 92.1% and 82.4%, respectively. 

### 2.2. White Blood Cell and Immunoglobulins Determination

Blood samples were stored at 4 °C and sent to the China Medical University Hospital for assaying WBC counts and the immunoglobulin in 24 h. The white blood cell counts were performed with flow cytometry (Automated Hematology Analyzer of Beckman Coulter LH series). A 10 mL blood sample was centrifuged to obtain serum for IgA and IgG assay using turbidimetry (Nephelometer, Hitachi 747, Tokyo, Japan), and IgE quantification using Enzyme-linked immunoassay [16].

### 2.3. Statistical Analysis

Data analysis first calculated mean (standard deviation, SD) levels of hair As, Cd and Pb for 133 male participants recruited from the 5 plants by age, type of plant they worked with, smoking and drinking. We further calculated the 25th, 50th and 75th percentile values of PB, Cd and As in hair samples. Mean values of White blood cell (WBC), lymphocyte, monocyte, IgG, IgA and IgE by levels of Pb, Cd and As in percentiles of <25th, 25th–74th and ≥75th were measured and tested using one-way ANOVA. We further calculated Spearman’s correlation coefficients between these immunological indicators and the 3 trace elements. 

## 3. Results

### 3.1. Mean Values of As, Cd and Pb by Age, Plant Type and Lifestyle

Mean values of As, Cd and Pb in hair samples decreased with age, the trend was significant for mean Pb levels (Table 1). Levels of Cd exposure also varied little in association with types of plant, smoking and drinking. The overall mean value was the highest for Pb and the lowest for Cd. Participants recruited from glass plants had higher exposure to trace elements. Mean hair levels of these elements were not higher in smokers. Drinking was associated with a slightly higher mean Pb level, but significant. 

### 3.2. The Quintile Values of As, Cd and Pb

Table 2 shows means of the 3 trace elements in hair samples and the 25th, 50th and 75th percentile levels among the participants. The levels changed greater for Pb and As than for Cd, from 0.74 µg/g at the 25th percentile to 1.11 µg/g at the 75th percentile for Pb and correspondingly from 0.74 µg/g to 0.91 µg/g for As. The Cd levels varied little among participants.

### 3.3. Associations betweem Immunological Indicators and Selected Metals in Hair Sample

Table 3 shows mean values of immunological indicators by the percentile ranges (<25th, 25th–74th and ≥75th) of trace elements. Measured mean values of all indicators were not different among the 3 Pb groups and among the 3 Cd groups. Mean IgG and IgE values decreased as the As level increased, and the relationship was significant between As and IgG (*p* = 0.014) and between As and IgE (*p* = 0.033). The mean IgA value was also the lowest for individuals with hair As at ≥75th levels (*p* = 0.012).

In the Spearman’s correlation analysis, results showed significant positive relationship between monocyte values and hair Pb levels, but negative relationships between IgG and As levels and between IgE and As levels (*p* < 0.05) (Table 4).

## 4. Discussion

In this study, we evaluated mean values of each immunologic factor by percentile levels of As, Cd and Pb. We found that only mean values of IgA and IgE in blood were significantly varied by the hair As level. Mean IgA was higher in men for the 25–74th percentile levels of As, while the mean IgE reduced as the As level increased. However, the Spearman’s correlation analysis showed that the hair As concentration was significantly reversed associated with the blood IgE value, but not with IgA values. It is interesting to note a significant positive relationship between blood monocyte values and hair Pb levels. 

Previous studies on the immunologic function associating with Cd are mainly animal models with effects varying by exposure levels of Cd [4]. Low levels of Cd exposure may enhance the humoral immune response, while high levels of exposure may reduce the antibody production. An animal study found that oral intake of 5 ppm and 50 ppm of Cd can cause gut tissue injury, stimulating the immune activities of mesenteric lymph node cells [3]. Exposure to such a high Cd concentration is generally not possible to occur in human life. However, analysis of the US NHANES data reveals that the susceptibility to chronic infections, including *Helicobacter pylori*, *Toxoplasma gondii* and Hepatitis B virus, increases as the blood Cd level increases from 13.4 μg/L in tertile 1 to 30.5 μg/L in tertile 3 [6]. Similar relationship has also been reported between blood Pb levels and these chronic infections in the NHANES data-based study. We were unable to identify any immunologic function in association with Cd exposure in our study. The Cd burden was the lowest with little variation among all participants in our study. The Cd exposure was not high enough to exert any impact. 

Lead is a toxic heavy metal that attracts great attention in its relations with neurological damage and hematological impact [17,18,19,20]. Individuals with elevated occupational exposure to Pb are at increased risk of hemolytic anemia. Kuo et al. found that the blood monocyte count was reduced in battery workers comparing to school teachers [21]. The blood Pb burden was much greater in battery workers than in teachers. The Pb exposure may also have a role in the risk of autoimmune diseases [22]. In contrast, our Spearman’s correlation analysis shows that the monocyte count increased as Pb burden increased. However, this relationship seems unconvincing as the mean monocyte was lower for those with medium lead exposure levels. The Pb burden in our study participants is lower than that in battery workers. It is not clear whether lower Pb burden stimulates the production of monocytes. Monocytes are a type of white blood cell produced by the bone marrow serving immune functions as phagocytosis, antigen presentation, and cytokine production. In our study, monocyte counts were not associated with the hair As levels. It is likely that the As burden is not large enough to affect.

Chronic exposure to arsenic in drinking water is a devastating problem for millions of people in Bangladesh. The plasma As levels in school children are positively associated with total IgG and IgE, but not IgA [23]. These relationships are more evident for boys than for girls, particularly for underweight children. In an earlier study also in Bangladesh, Islam et al. found increased serum total IgG, IgE and IgA levels in adults with skin lesion in arsenic endemic rural villages [24]. Arsenicosis patients with respiratory complications have elevated IgE levels as well. An India study found significantly decreased monocyte counts in arsenic-exposed individuals with severe dermatological manifestations [25]. Higher As exposure increases the risk of infection and inflammation, leading to more serious disorders [26]. A peripheral arterial disease known as Blackfoot disease endemic in southwestern Taiwan has been associated with chronic poisoning and results from the consumption of arsenic-rich underground water over long periods. Patients may suffer from complications of lower basal microcirculatory perfusion [27,28]. Severe cases may suffer from ulceration and gangrene, or surgical amputation for the lower extremities. These arsenicosis patients are at increased risk of not only cancers but also cardiovascular disorders [29,30,31,32,33]. Findings in the present study suggested that serum concentration of IgA, IgE and IgG were lower for the industrial male workers with higher hair As concentrations. Arsenic can also act as a stimulator of the immune system. This negative relationship was not significant for IgA in the Spearman correlation analysis.

Large numbers of people have been found to be exposed to low levels of heavy metals. Results from epidemiologic studies evaluating the relationships between the risk of immune dysfunction and these elements are conflicting [34,35,36,37,38]. Studies could be limited to cross-sectional designs with study population of low exposure levels. Findings in this study are also limited by the cross-sectional design with low burden of As, Cd and Pb, although the contents in the hair represent the historical exposure in recent months from not only the industry but also diet and drink. Further data analysis showed a strong correlation between As levels in air samples at work and in hair samples (correlation coefficient = 0.467, *p* < 0.001) (data not shown). Our findings in relations with immunologic indicators are not consistent with previous studies. Previous studies included individuals with high exposure or with apparent health disorders. Our study population may benefit with healthy worker effects with low burden of As, Cd and Pb. Studies with different population groups are needed to confirm our findings.

## 5. Conclusions

This study used hair samples to measure the historical exposure to trace elements of industrial employees. However, we were unable to evaluate the impact of Cd because of low levels. As levels in hair were negatively associated with IgG and with IgE. However, whether As levels in hair represent a stronger relation with immunologic function than Cd and PB remains unclear. In the quest to evaluate the impact of As, Cd and Pb of low human burden, we need data with a larger range of concentrations from a much larger survey.

## Figures and Tables

**Table 1:** Mean levels of As, Cd and Pb in hairs of study subjects (n = 133) by their age, job type and lifestyle.

Personal Characteristics	n	Metal in Hair		
		Pb (μg/g)	Cd (μg/g)	As (μg/g)
		Mean (SD) ^a^	Mean (SD) ^a^	Mean (SD) ^a^
Age, Years		0.91 (0.22)	0.16 (0.03)	0.86 (0.16)
<30	16	1.10 (0.14)	0.18 (0.02)	0.92 (0.16)
30–50	90	0.89 (0.22)	0.16 (0.03)	0.86 (0.17)
>50	27	0.85 (0.20)	0.17 (0.03)	0.83 (0.14)
*p*-value *		<0.001	0.22	0.26
Factory			
Glass	75	1.02 (0.22)	0.17 (0.03)	0.89 (0.19)
Antimony	23	0.82 (0.11)	0.16 (0.03)	0.87 (0.15)
Plastics	35	0.72 (0.08)	0.16 (0.03)	0.81 (0.07)
*p*-value *		<0.001	0.12	0.04
Smoking			
No	94	0. (0.23)	0.15 (0.02)	0.85 (0.16)
Yes	39	0.89 (0.19)	0.19 (0.03)	0.88 (0.17)
*p*-value *		0.56	0.15	0.33
Drinking			
No	89	0.88 (0.20)	0.16 (0.03)	0.86 (0.16)
Yes	44	0.97 (0.24)	0.17 (0.03)	0.86 (0.17)
*p*-value *		0.01	0.21	0.92

^a^ SD: standard deviation; * Kruskal–Wallis Test or Mann–Whitney U test.

**Table 2:** The Distribution of Pb, Cd and As in hair of study population (n = 133).

Metal in Hair	Percentile			Mean (SD) ^a^
	25th	50th	75th	
Pb (µg/g)	0.74	0.87	1.11	0.91 (0.22)
Cd (µd/g)	0.14	0.15	0.18	0.16 (0.03)
As (µg/g)	0.76	0.82	0.91	0.86 (0.16)

^a^ SD: standard deviation.

**Table 3:** Mean levels of immunological indicators by quartile distributions of metals in hair samples.

Immunological Indicators	Metal in Hair			*p*-Value ^b^
	<25th Tile	25th–74th Tile	≥75th Tile	
	Mean (SD) ^a^	Mean (SD) ^a^	Mean (SD) ^a^	
Pb in hair				
WBC *, 10^3^/μL	6.43 (2.38)	6.38 (1.81)	6.42 (1.67)	0.992
Lymphocyte, %	31.97 (5.22)	32.28 (4.37)	33.69 (4.15)	0.222
Monocyte, %	6.73 (0.93)	6.67 (0.84)	6.85 (0.46)	0.530
IgG, mg/dL	966.7 (113.0)	944.1 (131.7)	928.2 (121.8)	0.440
IgA, mg/dL	240.1 (40.8)	231.2 (31.4)	228.3 (25.4)	0.293
IgE, mg/dL	127.8 (23.8)	128.1 (16.4)	127.3 (18.9)	0.809
Cd in hair				
WBC *, 10^3^/μL	6.02 (1.55)	6.37 (1.89)	6.68 (2.13)	0.420
Lymphocyte, %	33.50 (3.45)	32.84 (4.57)	31.66 (4.98)	0.247
Monocyte, %	6.72 (0.47)	6.69 (0.81)	6.82 (0.87)	0.670
IgG, mg/dL	949.6 (112.7)	955.4 (130.2)	926.1 (121.3)	0.484
IgA, mg/dL	232.1 (28.1)	237.5 (29.7)	224.6 (38.3)	0.129
IgE, mg/dL	125.1 (17.2)	127.6 (16.9)	128.2 (22.9)	0.822
As in hair				
WBC *, 10^3^/μL	6.23 (1.57)	6.16 (1.78)	7.07 (2.34)	0.064
Lymphocyte, %	32.69 (3.27)	32.60 (4.91)	32.47 (4.98)	0.981
Monocyte, %	6.88 (0.75)	6.60 (0.73)	6.86 (0.88)	0.146
IgG, mg/dL	963.2 (106.6)	955.1 (137.9)	909.7 (107.5)	0.014
IgA, mg/dL	233.4 (20.1)	239.2 (36.3)	218.9 (31.0)	0.012
IgE, mg/dL	131.2 (17.5)	129.1 (14.9)	120.2 (24.9)	0.033

^a^ SD: standard deviation; ^b^ test by One-Way ANOVA; * White blood cell.

**Table 4:** Spearman’s correlation between immunological indicators in blood samples and arsenic, cadmium and lead levels in hair samples.

Immunological Indicators	Metal in Hair		
	Pb	Cd	As
WBC	0.089	0.071	0.131
Lymphocyte	0.113	−0.111	0.027
Monocyte	0.185 *	0.079	−0.013
IgG	−0.128	−0.023	−0.184 *
IgA	−0.132	−0.091	−0.098
IgE	−0.109	0.110	−0.176 *

* *p* < 0.05.

## References

[1] Soto-Peña G.A., Luna A.L., Acosta-Saavedra L., Conde P., López-Carrillo L., Cebrián M.E., Vega L. (2006). Assessment of lymphocyte subpopulations and cytokine secretion in children exposed to arsenic. FASEB J..

[2] Wang Y., Zhao H., Shao Y., Liu J., Li J., Xing M. (2017). Copper or/and arsenic induce oxidative stress-cascaded, nuclear factor kappa B-dependent inflammation and immune imbalance, trigging heat shock response in the kidney of chicken. Oncotarget.

[3] Ninkov M., Aleksandrov A.P., Demenesku J., Mirkov I., Mileusnic D., Petrovic A., Brceski I. (2015). Toxicity of oral cadmium intake: Impact on gut immunity. Toxicol. Lett..

[4] Descotes J. (1992). Immunotoxicology of cadmium. IARC Sci. Publ..

[5] Dietert R.R., Piepenbrink M.S. (2006). Lead and immune function. Crit. Rev. Toxicol..

[6] Krueger W.S., Wade T.J. (2016). Elevated blood lead and cadmium levels associated with chronic infections among non-smokers in a cross-sectional analysis of NHANES data. Environ. Health.

[7] Van DenBerg K.R., Freeborn R.A., Liu S., Kennedy R.C., Zagorski J.W., Rockwell C.E. (2017). Inhibition of early T cell cytokine production by arsenic trioxide occurs independently of Nrf2. PLoS ONE.

[8] Zhang X., Su Y., Zhang M., Sun Z. (2012). Opposite effects of arsenic trioxide on the Nrf2 pathway in oral squamous cell carcinoma in vitro and in vivo. Cancer Lett..

[9] Başaran N., Ündeğer Ü. (2000). Effects of lead on immune parameters in occupationally exposed workers. Am. J. Ind. Med..

[10] Wu C.C., Chen Y.C. (2017). Assessment of Industrial Antimony Exposure and Immunologic Function for Workers in Taiwan. Intern. J. Environ. Res. Pub. Health.

[11] Foo S.C., Khoo N.Y., Heng A., Chua L.H., Chia S.E., Ong C.N., Ngim C.H., Jeyaratnam J. (1993). Metals in hair as biological indices for exposure. Int. Arch. Occup. Environ. Health.

[12] Kempson I.M., Lombi E. (2011). Hair analysis as a biomonitor for toxicology, disease and health status. Chem. Soc. Rev..

[13] Niculescu T., Dumitru R., Botha V., Alexandrescu R., Manolescu N. (1983). Relationship between the lead concentration in hair and occupational exposure. Br. J. Ind. Med..

[14] Seidel S., Kreutzer R., Smith D., McNeel S., Gilliss D. (2001). Assessment of commercial laboratories performing hair mineral analysis. JAMA.

[15] Goulle J.P., Mahieu L., Castermant J., Neveu N., Bonneau L., Laine G., Bouige D., Lacroix C. (2005). Metal and metalloid mult-elementary ICP-MS validation in whole blood, plasma, urine and hair. Reference values. Forensic Sci. Int..

[16] Talarmin A., Labeau B., Lelarge J., Sarthou J.L. (1998). Immunoglobulin A-specific capture enzyme-linked immunosorbent assay for diagnosis of dengue fever. J. Clin. Microbiol..

[17] Baghurst P.A., McMichael A.J., Wigg N.R., Vimpani G.V., Robertson E.F., Roberts R.J., Tong S.L. (1992). Environmental exposure to lead and children’s intelligence at the age of seven years: The Port Pirie Cohort Study. N. Engl. J. Med..

[18] Bellinger D.C., Stiles K.M., Needleman H.L. (1992). Low-level lead exposure, intelligence and academic achievement: A long-term follow-up study. Pediatrics.

[19] US Department of Health and Human Services (2007). Agency for Toxic Substances and Disease Registry: Toxicological profile for Lead.

[20] Hsieh N.H., Chung S.H., Chen S.C., Chen W.Y., Cheng Y.H., Lin Y.J., Liao C.M. (2017). Anemia risk in relation to lead exposure in lead-related manufacturing. BMC Public Health.

[21] Kuo H.W., Hsiao T.Y., Lai J.S. (2001). Immunological effects of long-term lead exposure among Taiwanese workers. Arch. Toxicol..

[22] Mishra K.P. (2009). Lead exposure and its impact on immune system: A review. Toxicol. In Vitro.

[23] Raqib R., Ahmed S., Ahsan K.B., Kippler M., Akhtar E., Roy A.K., Lu Y., Vahter M. (2017). Humoral Immunity in Arsenic-Exposed Children in Rural Bangladesh: Total Immunoglobulins and Vaccine-Specific Antibodies. Environ. Health Perspect..

[24] Islam L.N., Nurun Nabi A.H.M., Rahman M.M., Zahid M.S.H. (2007). Association of respiratory complications and elevated serum immunoglobulins with drinking water arsenic toxicity in human. J. Environ. Sci. Health Part A.

[25] Maiti S., Chattopadhyay S., Deb B., Samanta T., Maji G., Pan B., Ghosh A., Ghosh D. (2012). Antioxidant and metabolic impairment result in DNA damage in arsenic-exposed individuals with severe dermatological manifestations in Eastern India. Environ. Toxicol..

[26] Ferrario D., Gribaldo L., Hartung T. (2016). Arsenic Exposure and Immunotoxicity: A Review Including the Possible Influence of Age and Sex. Curr. Environ. Health Rep..

[27] Tseng W.P. (1977). Effects and dose—Response relationships of skin cancer and blackfoot disease with arsenic. Environ. Health Perspect..

[28] Tseng C.H. (2006). Blackfoot Disease and Arsenic: A Never-Ending Story. J. Environ. Sci. Health Part C.

[29] Chiou H.Y., Chiou S.T., Hsu Y.H., Chou Y.L., Tseng C.H., Wei M.L., Chen C.J. (2001). Incidence of transitional cell carcinoma and arsenic in drinking water: A follow-up study of 8102 residents in an arsenic-endemic area in Northeastern Taiwan. Am. J. Epidemiol..

[30] Cheng P.S., Weng S.F., Chiang C.H., Lai F.J. (2016). Relationship between arsenic-containing drinking water and skin cancers in the arseniasis endemic areas in Taiwan. J. Dermatol..

[31] Lin H.J., Sung T.I., Chen C.Y., Guo H.R. (2013). Arsenic levels in drinking water and mortality of liver cancer in Taiwan. J. Hazard Mater..

[32] Chen Y.T., Wu C.C., Liu X.P., Chai C.Y. (2017). Aberrant β-catenin expression in urothelial carcinomas in blackfoot disease-endemic areas. Kaohsiung J. Med. Sci..

[33] Chou Y.L., Chiou S.T., Hsueh Y.M., Chiou H.Y., Chen C.J. (2001). A study on the association between carotid atherosclerosis and genetic polymorphisms of apolipoprotein E among various arsenic exposure people. Taiwan J. Public Health.

[34] Koller L.D. (1980). Immunotoxicology of heavy metals. Int. J. Immunopharmacol..

[35] Selgrade M.K. (2007). Immunotoxicity: The risk is real. Toxicol. Sci..

[36] Vas J., Monestier M. (2008). Immunology of mercury. Ann. N. Y. Acad. Sci..

[37] Hanson M.L., Holaskova I., Elliott M., Brundage K.M., Schafer R., Barnett J.B. (2012). Prenatal cadmium exposure alters postnatal immune cell development and function. Toxicol. Appl. Pharmacol..

[38] Dangleben N.L., Skibola C.F., Smith M.T. (2013). Arsenic immunotoxicity: A review. Environ. Health.

